# Melt Analysis of Mismatch Amplification Mutation Assays (Melt-MAMA): A Functional Study of a Cost-Effective SNP Genotyping Assay in Bacterial Models

**DOI:** 10.1371/journal.pone.0032866

**Published:** 2012-03-16

**Authors:** Dawn N. Birdsell, Talima Pearson, Erin P. Price, Heidie M. Hornstra, Roxanne D. Nera, Nathan Stone, Jeffrey Gruendike, Emily L. Kaufman, Amanda H. Pettus, Audriana N. Hurbon, Jordan L. Buchhagen, N. Jane Harms, Gvantsa Chanturia, Miklos Gyuranecz, David M. Wagner, Paul S. Keim

**Affiliations:** 1 Center for Microbial Genetics and Genomics, Northern Arizona University, Flagstaff, Arizona, United States of America; 2 National Center for Disease Control and Public Health and Ilia State University, Tbilisi, Georgia, United States of America; 3 Translational Genomics Research Institute, Phoenix, Arizona, United States of America; 4 Veterinary Medical Research Institute, Hungarian Academy of Sciences, Budapest, Hungary; 5 Department of Veterinary Pathology, Western College of Veterinary Medicine, University of Saskatchewan, Saskatoon, Saskatchewan, Canada; Naval Research Laboratory, United States of America

## Abstract

Single nucleotide polymorphisms (SNPs) are abundant in genomes of all species and biologically informative markers extensively used across broad scientific disciplines. Newly identified SNP markers are publicly available at an ever-increasing rate due to advancements in sequencing technologies. Efficient, cost-effective SNP genotyping methods to screen sample populations are in great demand in well-equipped laboratories, but also in developing world situations. Dual Probe TaqMan assays are robust but can be cost-prohibitive and require specialized equipment. The Mismatch Amplification Mutation Assay, coupled with melt analysis (Melt-MAMA), is flexible, efficient and cost-effective. However, Melt-MAMA traditionally suffers from high rates of assay design failures and knowledge gaps on assay robustness and sensitivity. In this study, we identified strategies that improved the success of Melt-MAMA. We examined the performance of 185 Melt-MAMAs across eight different pathogens using various optimization parameters. We evaluated the effects of genome size and %GC content on assay development. When used collectively, specific strategies markedly improved the rate of successful assays at the first design attempt from ∼50% to ∼80%. We observed that Melt-MAMA accurately genotypes across a broad DNA range (∼100 ng to ∼0.1 pg). Genomic size and %GC content influence the rate of successful assay design in an independent manner. Finally, we demonstrated the versatility of these assays by the creation of a duplex Melt-MAMA real-time PCR (two SNPs) and conversion to a size-based genotyping system, which uses agarose gel electrophoresis. Melt-MAMA is comparable to Dual Probe TaqMan assays in terms of design success rate and accuracy. Although sensitivity is less robust than Dual Probe TaqMan assays, Melt-MAMA is superior in terms of cost-effectiveness, speed of development and versatility. We detail the parameters most important for the successful application of Melt-MAMA, which should prove useful to the wider scientific community.

## Introduction

Single nucleotide polymorphisms (SNPs) are point mutations with biological significance across diverse scientific disciplines ranging from medicine to agriculture. SNPs are useful in predicting the disposition for some diseases [1], [2], as indicators for the genetic basis for varying responses to pharmacological drug treatment [3], for classifying bacterial populations into specific genetic groups [4], [5], and for association with specific phenotypic traits, such as insecticide resistance in insects [6]. Technologies that permit cost-effective yet expeditious SNP interrogation are in demand.

Numerous SNP detection technologies have been developed over the past 20 years and have been extensively described in several published reviews [7]–[10]. Many of these technologies are based on real-time PCR. Real-time PCR instruments are present in many clinical and research laboratories because of their efficiency, automation, experimental simplicity, and amenability to high capacity throughput. Two of the more prevalent real-time technologies for SNP interrogation on these instruments are Dual Probe TaqMan and Allele-Specific (AS) PCR assays, each of which utilizes different genotyping strategies and material components.

Dual Probe TaqMan assays amplify the target amplicon, whereas the SNP locus is concurrently genotyped by one of the two allele-specific internal probes. These internal probes are differentially labeled with fluorescent dyes. For TaqMan assays, the specificity of the probe to an internal region of the amplicon confers superb detection of extremely low template amounts down to a single genomic equivalent [4], [5]. However, the fluorescently labeled internal probes make the cost of TaqMan assays approximately fourteen times higher than the cost of assays that are solely based on unlabeled primers, such as AS-PCR assays (https://products.appliedbiosystems.com) (https://www.idtdna.com). As a consequence, Dual Probe TaqMan assays are cost-prohibitive for laboratories with a limited budget or in studies interested only in small-scale SNP screening. In addition, the turn-around time required to synthesize and ship labeled internal probes takes approximately 7–10 days compared with only 2–3 days for unlabeled primers. This slower production rate of TaqMan probe assays may make them unfeasible in situations that demand rapid confirmation of newly identified SNPs.

SNP genotyping with an AS-PCR assay is achieved by two AS-forward primers that act in concert with a single reverse primer. The AS primer design introduces a mismatch at the 3′ end with a DNA template composed of a non-complementary SNP state (non-allelic template). This non-complementary base mismatch is not observed with an allelic (matched) template. The 3′ end mismatch decreases the extension efficiency of *Taq* polymerase by 15% to 50% per cycle [11] resulting in lower PCR efficiency when compared to the perfect primer/template complex [12]. When the two AS-specific primers compete for the same template, the perfect primer/template interaction out-competes the mismatch primer/template interaction due to its more rapid extension efficiency, causing this primer-template complex to dominate in the PCR [13], [14]. SNP genotyping in AS-PCR is further enhanced by the incorporation of a destabilizing point mutation at the penultimate (–2) or ante-penultimate (–3) base position of the forward AS primers [15]–[18]. The labeling of one of the AS-forward primer with a GC-clamp enables facile differentiation of AS-PCR products through melt-curve analysis [17], [19]–[21], which has been termed mismatch amplification mutation assay or Melt-MAMA [19], [22]. Although AS-PCR assays are cost-effective due to the exclusive use of unlabeled primers, it is a technology hampered by high assay failure rates of ∼50% [23]. In addition, considerable knowledge gaps in areas such as assay capacity, sensitivity, and limitations exist.

There is a need for cost-effective genotyping alternatives for which unlabeled primer technologies such as Melt-MAMA could easily satisfy [16]–[20], [22], [24], [25]. Thus, it is important to identify design principles that reduce traditionally high assay failure rates. A better understanding of the robustness and sensitivity of Melt-MAMA would also promote its maximal application. In this study, we present specific strategies that improved the success rate of first-pass Melt-MAMA design, providing rates comparable to the Dual Probe TaqMan assay. Although the information presented here is based on bacterial pathogens, MAMA technologies have been successfully applied to viruses [26], [27] and eukaryotic species, including humans [17], [18], [20]. Therefore, these guidelines can be employed by the broader scientific community to maximize successful application of the Melt-MAMA SNP genotyping technology on other organisms of interest.

## Results and Discussion

Molecular schemes for subtyping bacterial pathogens rely on detecting known DNA-based differences among isolates, such as SNPs. SNPs are the most common DNA difference found among bacterial strains, yet assaying for them in a cost-effective and reliable manner has been problematic. Here, we summarize our efforts in employing Melt-MAMA across multiple pathogenic bacterial species for SNP interrogation and the positive effects of specific strategies that maximized the successful application of this cost-effective technique. Several optimization strategies that, when used collectively, effectively improved the assay success rate at the first design attempt from ∼50% to ∼80%. In addition, we demonstrated the Melt-MAMA generated amplification products can be monitored with the even more cost-effective, although slower, approach of agarose-gel electrophoresis. We conclude that Melt-MAMA is a highly effective and robust SNP genotyping approach applicable to laboratories with varying economic resources.

**Table 1:** Melt-MAMAs targeting specific groups within eight pathogen species.

Bacterial Pathogen	%GC content	Genome size	Assay Success Rate^a^	Total Assays	Total assay success^b^	Failed^c^	Increased primer non GC-clamp^d^	Increased primer (GC-clamp)^d^
*Francisella tularensis*	30%	1.9 Mb	98%	51	50	1	24	2
*Bacillus anthracis*	35%	5.2 Mb	87%	23	20	3	11	0
*Yersina pestis*	47%	4.6 Mb	81%,100%^b^	22	22^b^	4	0	0
*Escherichia coli*	50%	5.0 Mb	83%	6	5	1	0	0
*Burkholderia pseudomallei*	68%	7.3 Mb	40%	10	4	6	0	0
*Burkholderia mallei*	68%	5.8 Mb	72%	25	18	7	12	0
*Brucella suis*	56%	3.2 Mb	83%	12	10	2	6	0
*Coxiella burnetii*	42%	2 Mb	66%,89%^b^	36	32	4	14	3

a First design attempt and altered primer ratio optimization.

b Success after combining first or second design attempts and altered primer ratio optimization.

c Failed after first design attempt.

d Assays that required altered primer concentration ratios.

### Melt-MAMAs are robust across genetically diverse genomes

Over the course of multiple studies, 185 SNPs from eight bacterial pathogen species, differing greatly with respect to sequence, genome size and %GC content (Table 1), were developed into Melt-MAMA genotyping assays. All listed pathogens, with the exception of *E. coli*, are federally designated as Select Agents because of their potential for bio weaponization. Information gained from molecular assays that enable rapid SNP genotyping is critical for forensic and public health investigative efforts and could also be used to shape the course of therapeutic treatments by monitoring the presence of mutations associated with drug-resistance.

### A ‘unique’ destabilizing mismatch mutation enhance allele specificity

**Figure 1:**
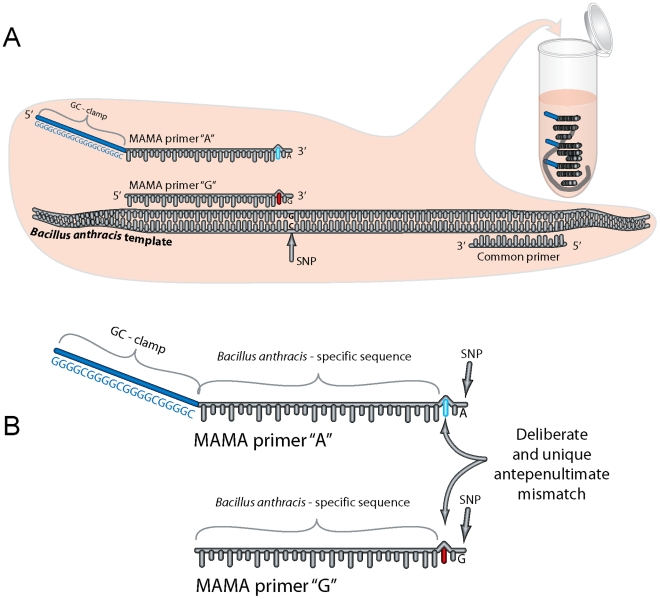
Melt-MAMA approach to SNP genotyping. A) Allele-specific (AS) primers are designed to detect the two alleles at a SNP locus on DNA template. These primers work in concert with a third common primer to generate a PCR amplicon. Depending on the template, only one of the AS primers will generate the amplicon in concert with the common primer. The ability to differentiate amplicon derived from each allele-specific primer is accomplished by the GC-clamp. The success of one versus the other is evaluated by melt-dissociation analysis, which is facilitated by the GC-clamp on the 5′ end of the one AS primer. B) Allele-specificity of primers is achieved by the incorporation of two independent design features that work nearly synergistically. First, the primer is designed in which the 3′ ultimate nucleotide directly overlaps the SNP locus in perfect complement. Second, allele-specificity is strengthened by engineering a mismatch at the antepenultimate position of the primer (MAMA primer) that uniquely differs in base composition to the template and alternate AS primer. This difference creates two mismatched nucleotides in the 3′ region of the primer for the non-allelic template, but only one difference in the correct allele template. This dramatically improves specificity by destabilizing the 3′ end of the non-allelic primer-template complex. Once a single round of PCR amplification has occurred, the antepenultimate difference is incorporated into the template for efficient subsequent rounds of amplification. Hence, the initial priming specificity is critical to accurate genotyping. Because the AS primers are added together in one reaction, they compete for priming, which also increases genotype fidelity over two independent reactions.

**Figure 2:**
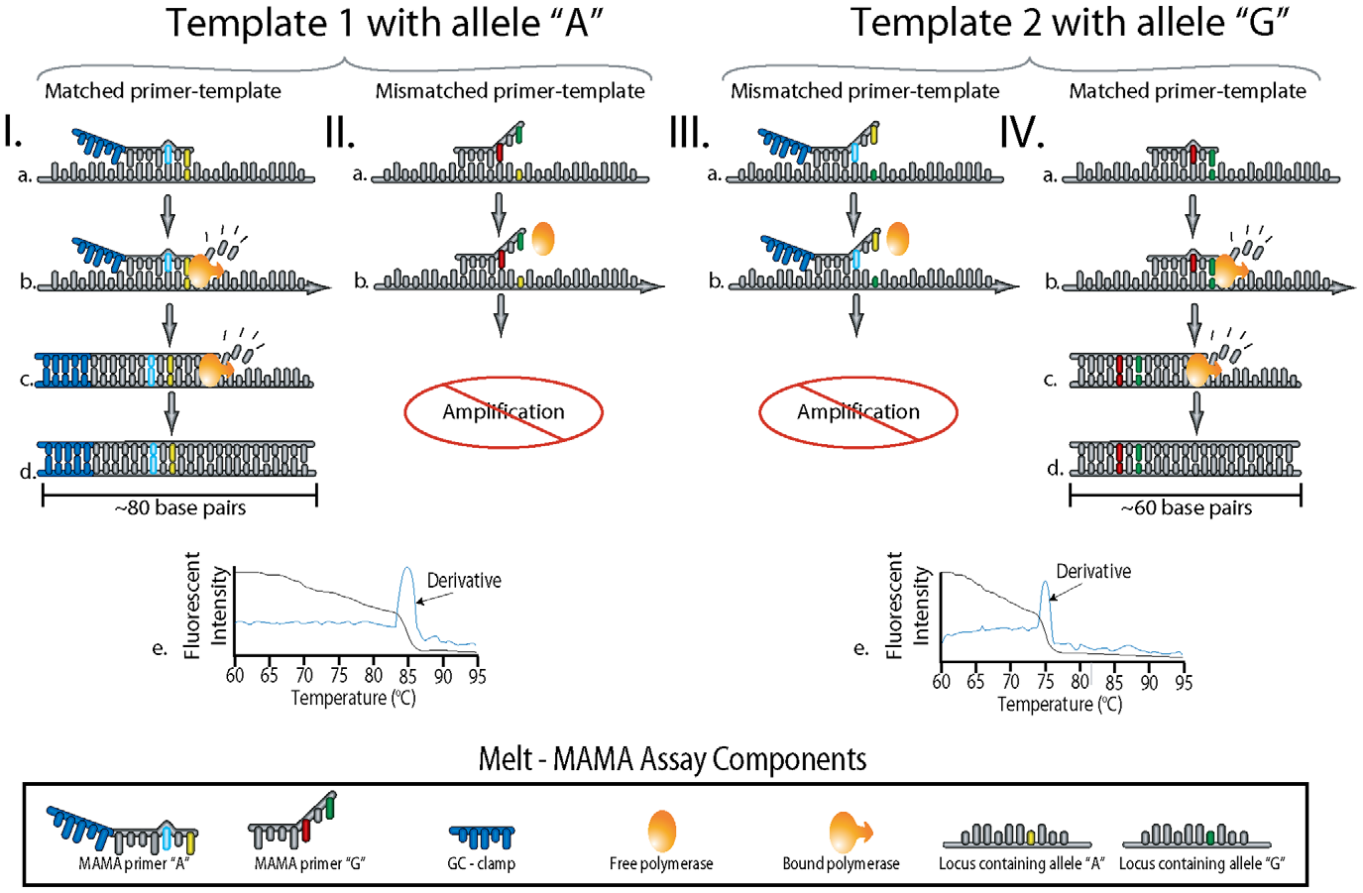
The principle of the Melt-MAMA PCR reaction. Four different scenarios involving two alternate SNP allele templates (I & II vs. III & IV) and the interaction of Allele-Specific (AS) PCR amplification using MAMA primers. The annealing of AS-MAMA primers to their allelic templates is shown with one primer labeled with a 5′ GC-clamp (Ia) whereas the other is not (IVa). (Ib and IVb) *Taq* Polymerase extends from the 3′ matched AS-MAMA primer despite the antepenultimate destabilizing nucleotide. (Ic and IVc) The second PCR cycle replicates from a newly synthesized DNA template made in the previous step (Ib and IVb). With the synthesized DNA serving as the template, a perfect primer-template complex is formed eliminating the antepenultimate destabilizing mismatch observed in Iab and IVab. At PCR endpoint (Id and IVd), the amplicons generated from the 3′ matched AS-MAMA primer greatly outnumbers the amplicons generated by the mismatched AS-MAMA primer. Temperature-dissociation curve plots (Ie and IVe) of each AS-PCR product (Iabcd, IIab and IIIab, IVabcd), showing the fluorescent intensity and the rate of fluorescent intensity change (derivative) as a function of temperature. For each allelic template reaction (I & II vs. III & IV), the melt profiles (Ie and IVe) show only a single change in fluorescent intensity. This indicates the amplification of the perfect-matched amplicon and little to no amplification of the mismatched amplicon. The GC –clamp “labeled” amplicons dissociate at higher temperatures (∼3°C to 5°C) than non-GC amplicons. Nonproductive primer annealing is shown for an AS-MAMA primer (IIa) and a GC-clamp AS-MAMA primer (IIIa) binding with their respective corresponding mismatched templates. The lack of Watson-Crick base pairing at two 3′ positions (the antepenultimate nucleotide at the 3′ end) of the AS primer introduces instability at this region (IIb and IIIb). This prevents efficient extension by the polymerase, which retards or prevents product amplification (Ie and IVe).

The SNP-allele discrimination of a MAMA is greatly enhanced by the design of an additional mismatch engineered at the penultimate or antepenultimate base position of the primer [15]–[17] (Figure 1). This mismatch strategy generates primer-template complexes containing a single- or double-base mismatch at the 3′ end of the primer (Figure 2). This capitalizes on the principle that primers with mismatch(es) at the 3′-end tend to “destabilize” primer-template annealing at mismatched sites and, therefore, will result in lower PCR efficiencies due to decreased extension efficiency of *Taq* polymerase [12] (Figure 2). In principle, the single mismatched primer-template complex is much more efficient than the double mismatched complex and, consequently, this efficiency differential permits the single mismatched primer to dominant the PCR (Figure 2I & IV).

It is imperative that Melt-MAMAs retain the two-nucleotide mismatch at the 3′ end of the non-complementary AS-MAMA primers after the initial cycle when the newly synthesized “nascent” DNA template becomes the dominant PCR template (Figure 2Ic & IVc). This feature retains the efficiency differential between the two competing AS-MAMA primers throughout the PCR. We designed all of our AS-MAMA primers to have destabilizing mismatch mutations with a nucleotide base that was uniquely different between the primer pairs (Figure 1 & 2) and the template DNA. Design consideration for the mismatch position penultimate or antepenultimate (–2 or –3, respectively) for each assay was based on achieving a unique nucleotide base at the mismatch positions while avoiding secondary structures within/among assay primers. We observed no advantage in assay function based on whether the mismatch position is –2 or –3 from the 3′-end, as previously observed [28]. Our experiments have shown that retaining this two-nucleotide mismatch strategy throughout the entire PCR enhanced SNP discrimination by minimizing the amplification via the non-complementary primer (data not shown).

Li et al. identified optimal two-nucleotide mismatch combinations for all SNP polymorphic states based on non-competitive TaqMan MAMA, where the AS-MAMA primers were separated into two individual reactions [16]. A small subset of combinations contained the same mutation at the mismatch positions for both AS-MAMA primers, a logical strategy from studies based on non-competitive MAMA [16]. However, these ideal combinations may not hold true under actual assay conditions where both AS-MAMA primers compete for the same DNA template, as in the Melt-MAMA single tube format (Figure 1). In such competitive assay conditions, the two-nucleotide mismatch advantage will be lost if the destabilizing mutation is not different from the DNA template and its alternate AS-MAMA primer. A small study of 12 *F. tularensis*-specific Melt-MAMAs, designed according to Li et al guidelines, resulted in a 41.6% initial success rate. This rate is slightly less than the ∼50% initial success rate of Melt-MAMAs designed without implementing the guidelines. These similar results between the two approaches suggest that the Li et al guidelines offer no advantage to competitive Melt-MAMAs and may even slightly reduce assay success rates.

**Figure 3:**
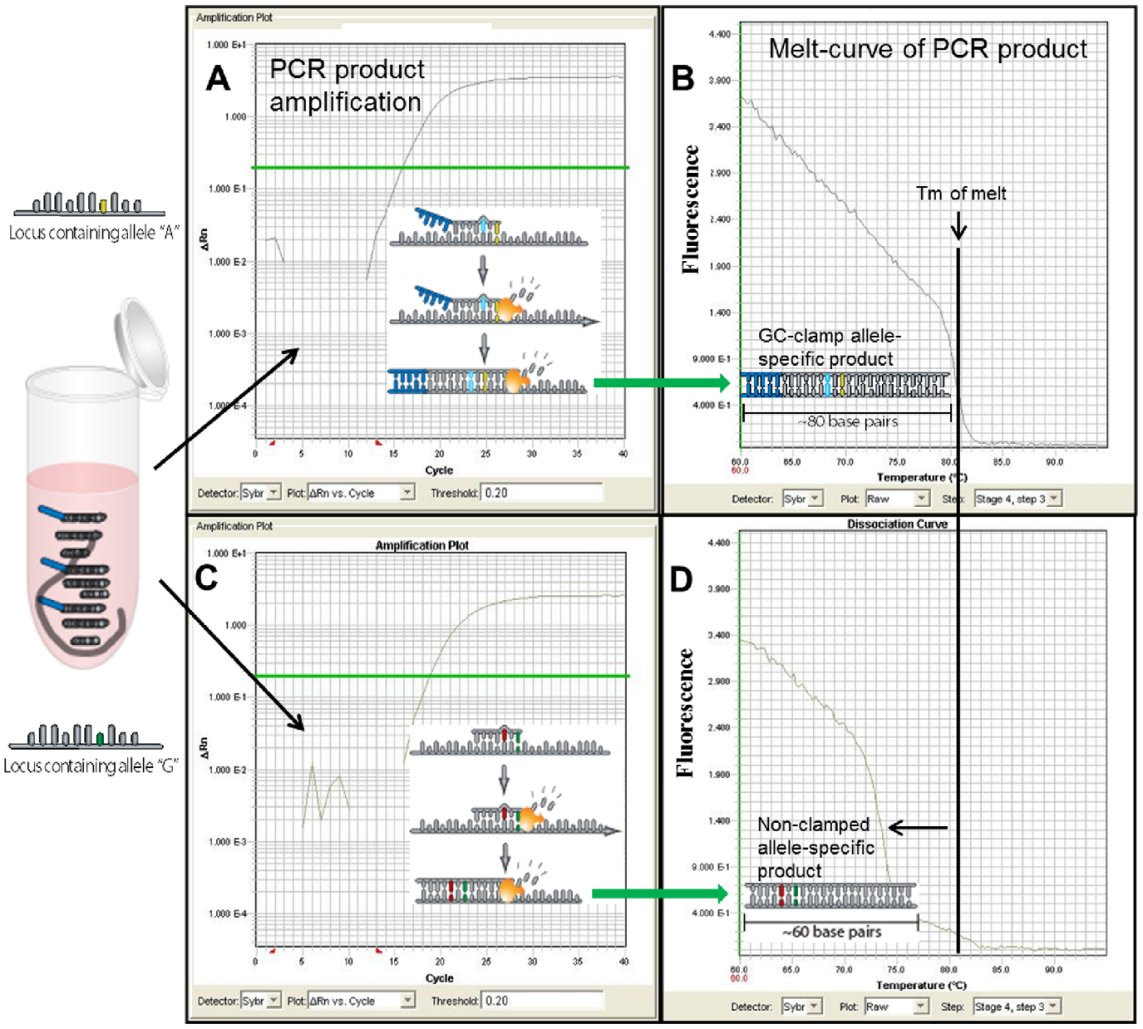
Real-time PCR amplification and dissociation (melt) curve plots. *B. anthracis* Melt-MAMA SYBR® Green assay targeting the A.Br.004 genetic clade. (A & C) The amplification of two alleles are illustrated for haploid template (*Bacillus anthracis*) possessing an ‘A’ polymorphic SNP-state or ‘G’ state. Each amplification plot represents a single PCR reaction containing a reverse “common” primer and two allele-specific MAMA primers. The AS-MAMA primers anneal to the same template target and then compete for extension across the SNP position. The polymerase-mediated extension rate of the 3′match AS-MAMA primer (perfect primer-template complex) exceeds that of the 3′mismatched MAMA primer (mismatched primer-template complex), thus the perfect match primer-template complex outcompetes the mismatched primer-template complex and dominates the PCR amplification. (B & D) Plots of the temperature-dissociation (melt) curve of the final PCR products for the two allele templates are shown next to their respective amplification plots (green arrows). Allele-specific PCR products are easily differentiated through temperature-dissociation (melt) curve analysis, which is conferred by the GC-clamp engineered on one of the AS-MAMA primer.

### Successful assay optimization by altering the primer ratios

**Figure 4:**
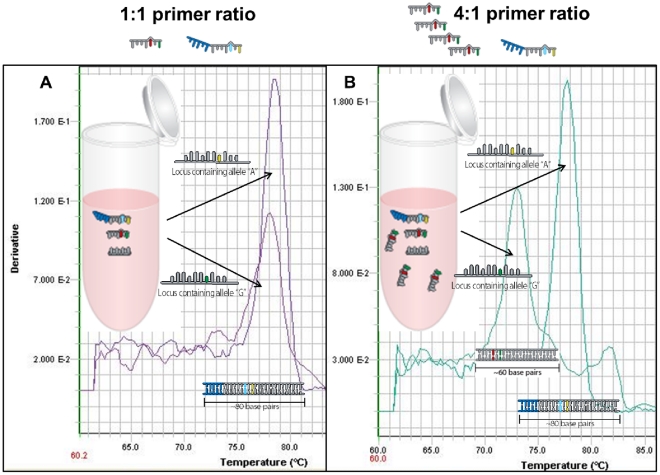
Assay optimization by altering the primer ratios. Primer design does not always result in perfectly matched combinations and additional improvement can be achieved by altering primer ratios. The derivative plots of the temperature-dissociation (melt) curves of two PCR products amplified in a *F. tularensis* assay are shown. Each product was amplified from genomic template with one of the two allele SNP-states (A or G). A) Under equal primer concentrations, this Melt-MAMA mis-genotyped the ‘G’ allele gDNA template because the G specific primer was more efficient than the A specific primer. In this case, the mismatched primer for the ‘G’ SNP allele state outcompeted the perfect matched primer, resulting in the amplification of the incorrect allele-specific PCR product. B) Primer ratios were then altered so that the matched primer for the “G’ SNP state was four times more concentrated than the respective mismatched primer. Under these unequal primer concentrations, the ‘G’ allele gDNA template accurately genotyped without the disruption of the accurate genotyping functions of the ‘A’ allele gDNA template.

**Figure 5:**
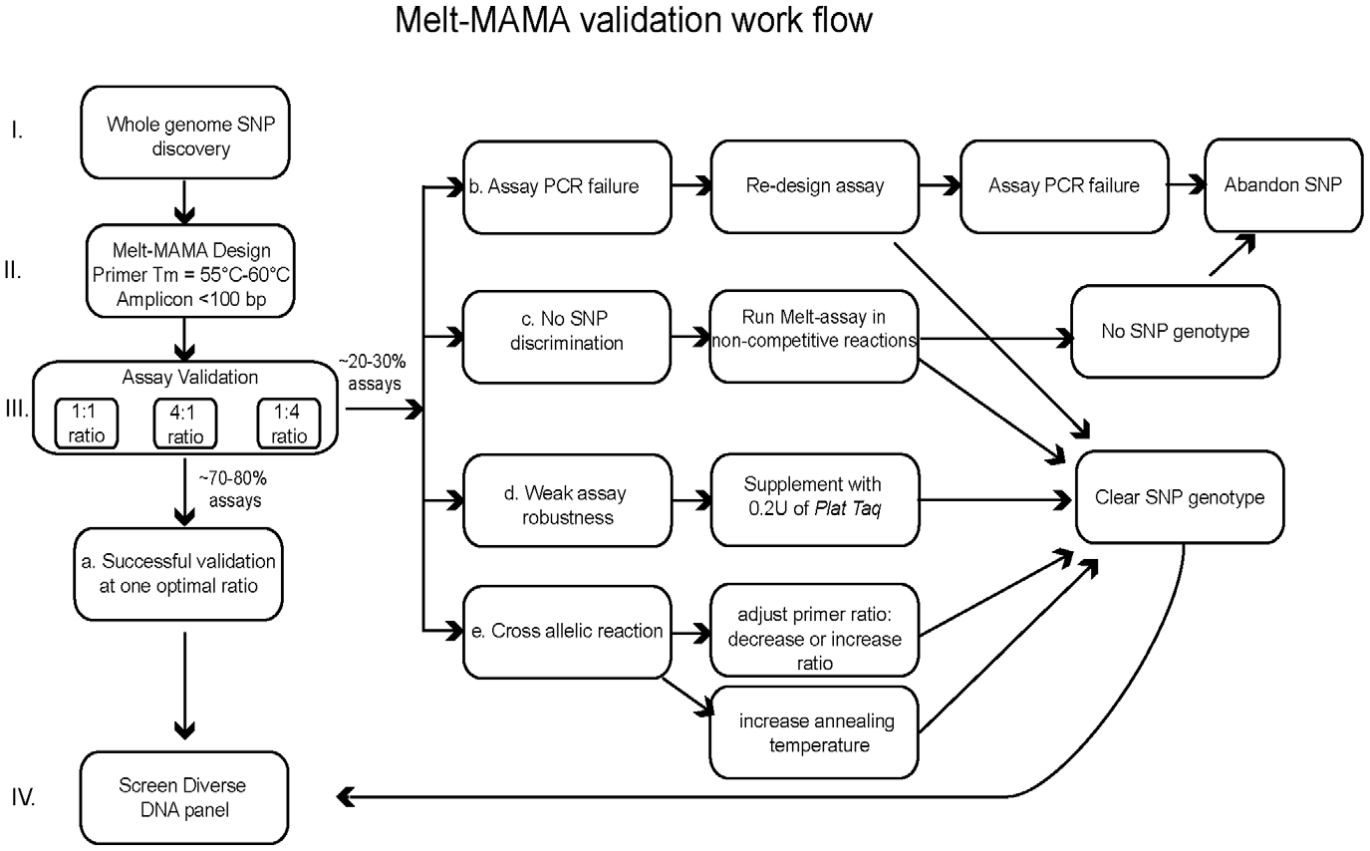
Melt-MAMA validation work flow. This figure shows the sequential steps involved in validation of Melt-MAMA assays. After SNP selection (step I), Melt-MAMA are designed so that the amplicon is <100 bp in length (step II). Assays are screened across ancestral and derived DNA templates under 3 primer ratio conditions where 1∶1 represents equal primer ratio, 4∶1 represents ancestral primer 4x and derived primer 1x, and 1∶4 represents ancestral primer 1x and derived primer 4x (step III). Five outcomes are indicated (step III a–e). Based on the performance of *B. anthracis*, *F. tularensis*, and *Y. pestis* assays, 70–80% Melt-MAMAs accurately genotyped at one of the tested primer ratio condition (step IIIa). These successful assays were immediately screened on a diversity panel of DNA samples (step IV). The remaining assays (20–30%) resulted in one of the other four outcomes (step III b–e). Each outcome required additional specific validation steps to determine the optimal PCR conditions or the need to abandon the SNP altogether. Our overall design success rate increased from 46% to 87%.

Despite the enhanced allele specificity endowed by the destabilizing mismatch design [15], our Melt-MAMAs often encountered costly design failure rates using equal primer concentrations. Accurately genotyping Melt-MAMAs succeed because the two AS-MAMA primers amplify only their respective allelic templates by out-competing the non-allelic alternate AS-MAMA primer (Figures 2 & 3). Although ∼45% of our assays behave in concordance to this prediction when at equal primer concentrations, our studies also show that equal primer stoichiometry caused ∼30% of our assays to perform poorly. Among the 185 Melt-MAMAs reported here, 72 assays displayed mild to severe cross-allele primer hybridization and resulted in poor (viewed as two melt profiles for a single DNA template; data not shown) to inaccurate allele discrimination by at least one of the AS-MAMA primers (Figure 4A). To rescue these poor performing assays, the concentration of the AS-primers were altered such that the concentration of the problematic allele-specific MAMA primer was less than the alternate “weaker performing” allele-specific MAMA primer by 2x, 3x, or 4x (See Methods; Figure 4B). The degree of reduction of the problematic AS-MAMA primer depended on the severity of the cross-reactivity. This strategy of altering primer concentrations resulted in accurate genotyping for both allelic templates (Figure 4B) in all 72 cases. Unexpectedly, we observed a pattern in which the more efficient AS-MAMA primer (67 out of 72) was the one labeled with the GC-clamp (Figure 4A). Additional validation studies across a known panel of diverse samples that represent each allele further demonstrated the fidelity of these “altered primer” assays (data not shown). Following a standard assay validation strategy that includes conditions of altered primer ratios (Figure 5), increased our total assay design success rate from 46% to 87% (Table 1). Twenty-eight other assays failed for miscellaneous reasons and could not be corrected by altering the primer ratio. This final category of SNP assays required more dramatic redesign strategies, such as primer design from the opposite strand, or abandonment of the SNP locus. However, abandonment was rare and, in our hands, Melt-MAMA success was comparable and possibly superior to the successful design rate of Dual Probe TaqMan assays, which also suffer from SNP locus design constraints [23].

As the design of two-nucleotide mismatch feature alone is not always sufficient to create the efficiency differential required for SNP discrimination at equal primer concentrations, Melt-MAMA performance is not solely determined by mismatches at the 3′-end. This finding has been previously reported [29], [30]. We observed that the presence of the GC-clamp appeared to influence the efficiency differential of Melt-MAMAs. The GC-clamp disproportionately favored higher efficiency (67 out of 72 assays) when primers were at equal concentrations while matched with the non-allelic DNA template. Taken together, these results suggest that the efficiency differential required for successful Melt-MAMAs is a product of both the nature of the mismatch at the 3′ end and the sequence of the entire primer. The GC-clamp appears to have a net effect of increasing the efficiency of many, but not all, assays. The exact mechanism for this observation is not well understood, could be multi-factorial, and was not tested in this study.

Allele-Specific PCR (AS-PCR) has been slow to gain popularity perhaps due to the ∼50% failure rate reported by others [12], [23] and observed in our study when assays are not further optimized by altering primer concentrations (Table 1). Chemically modifying primers to overcome this traditionally high rate of AS-PCR assay failure is an effective but expensive strategy [31]–[33]. Primer design guidelines to maximize the specificity of AS-MAMA primers for non-competitive MAMAs exist [16], but our experiments suggest that these guidelines are poorly relevant to competitive Melt-MAMAs. Altering primer concentrations to generate efficiency differentials between AS-MAMA primers when competing for the same template have been used to optimize assays in other studies [29] and have been used with Dual Probe TaqMan assays to rescue poorly discriminating assays (data not shown). This simple strategy corrected assays that suffered from severe primer cross-allele genotyping and markedly improved our initial assay-design success rate. Our success rate is now comparable to Dual Probe TaqMan assays [23]. Anecdotally, more experienced assay design teams frequently exceed this assay-design success rate and we have achieved near 100% success on some projects. Our overall rate of 87% is a conserved program-wide rate. On specific projects where multiple design attempts were made, we were able to achieve an overall assay success rate of ≥90%.

### The effects of genome size and %GC content on assay-design success rate

We also investigated the effects of genome size (1.9 Mb–7.3 Mb) and %GC content (30%–68%) of the eight pathogen species to assess the success rate of Melt-MAMA development (Table 1). Our results demonstrated that assay-design success rate can be affected by these two genomic variables. Species with small genomes and low GC content, such as *Francisella tularensis* (1.9 Mb; 30% GC), had a markedly higher success rate of assay development than pathogens with large genomes and high GC content, such as *Burkholderia pseudomallei* (7.3 Mb; 68% GC) (98% and 40% success, respectively; Table 1). The negative impact of large genome size on assay success rate was illustrated by the comparison of the genomes of *Burkholderia pseudomallei* (7.3 Mb) and *Burkholderia mallei* (5.8 Mb). Despite their identical 68% GC content, assay success rates for these species markedly differed (40% and 72%, respectively; Table 1). Higher %GC content also appeared to negatively impact the success rate as illustrated by the comparison of the genomes *F. tularensis* (30% GC) and *Coxiella burnetii* (42% GC). Despite comparable genome size (1.9 Mb and 2 Mb, respectively), their assay-design success rates moderately differed (98% and ∼89%, respectively). These results suggest that the two independent variables of genome size and %GC content each contribute to assay success rates. The final assay-design success rate of the four remaining pathogens with mid-size genomes (3.2 Mb–5.2 Mb) and moderate %GC content (35%–56%) varied (83%–100%). Taken together, these results suggest that the final assay-design success rate of Melt-MAMAs is influenced by the genomic size and %GC content of the target organism in an independent and weighted manner. These observations are consistent with publications reporting that organisms with large genome size and high GC content tend to have poorer rates of successful PCR amplification [34]–[37]. That said, numerous publication on the MAMA technology used human genomes [17], [18], [20], which is large in size.

### Melt-MAMAs are not suitable for genotyping extremely low level DNA template amounts

**Figure 6:**
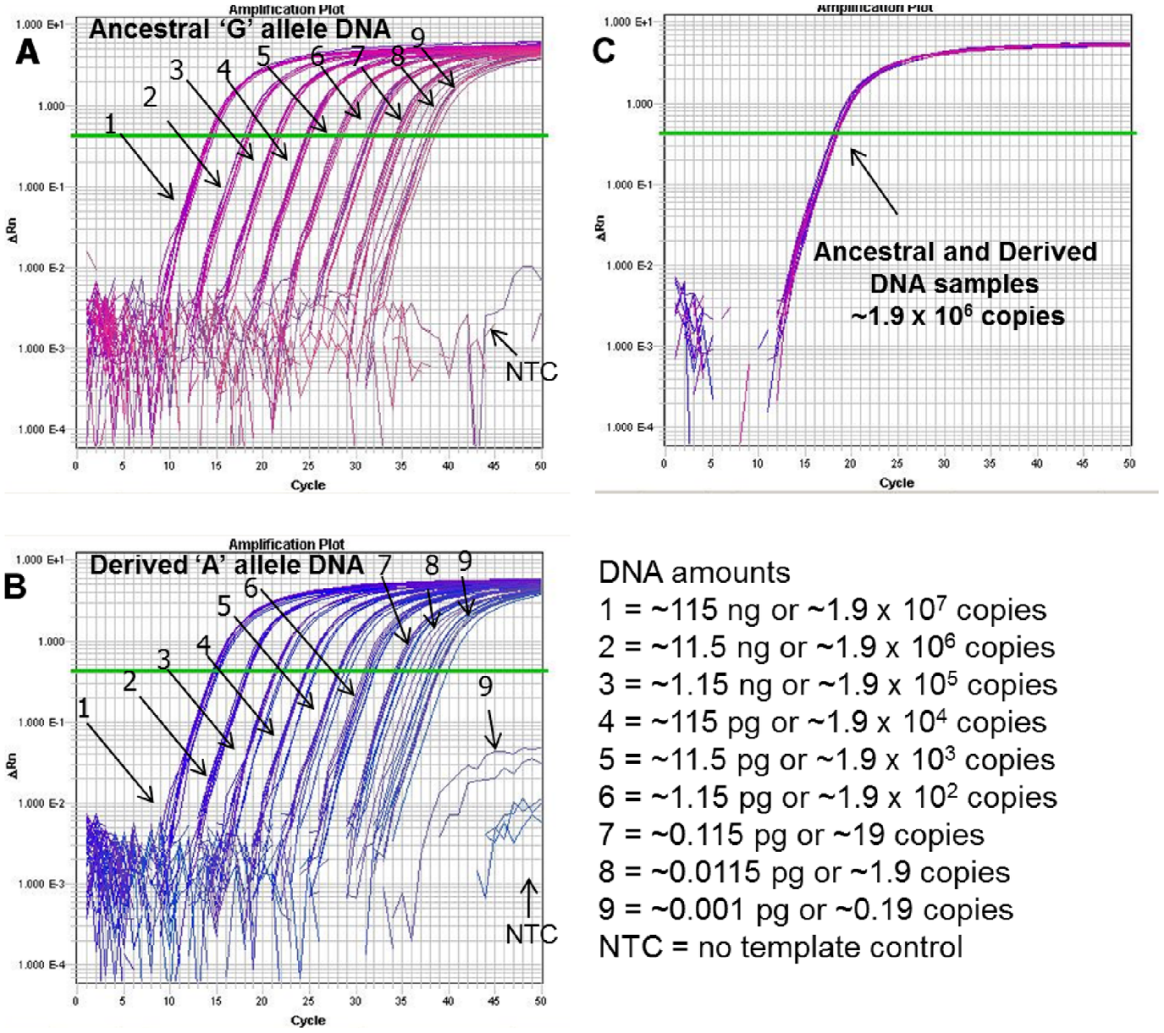
TaqMan assay performance at a broad range of DNA amounts. A *B. anthracis* TaqMan assay was used to screen the polymorphic ‘G’ or ‘A’ DNA templates (ancestral and derived, respectively) used in the *B. anthracis* Melt-MAMAs. (A & B) The respective amplification plots of genomic DNA of ‘G’ allele and ‘A’ SNP allele templates show the amplification curves of templates titrated in ten-fold serial dilutions and in replicates of eight. The number assigned to each amplification curve (1–9) denotes the DNA amount for the starting template. (C) Both genomic template types were of equal amounts. The consistency of amplification dropped with lower amounts of initial template, but the dilution levels containing less than a single copy (*B. anthracis* single copy ∼6 fg) was still detectable in some reactions. Detection of low-level DNA template by TaqMan assays is subject to stochastic sampling effects, which is predictable using a Poisson distribution [4].

**Figure 7 pone-0032866-g007:**
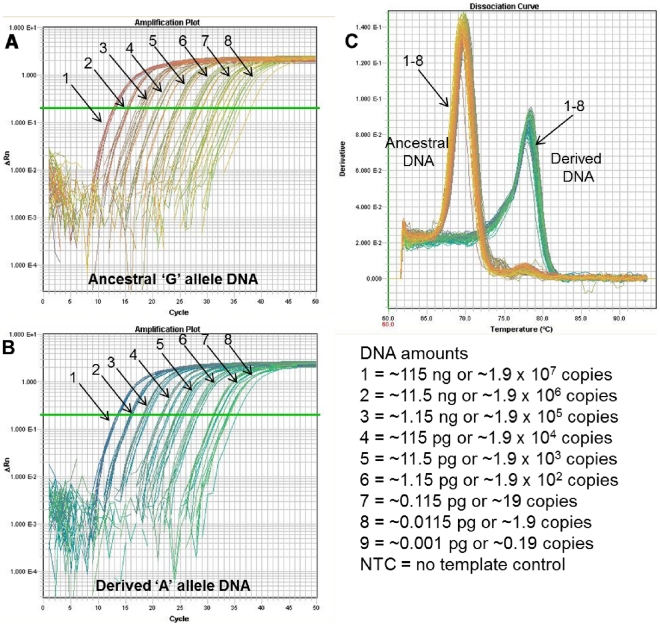
Genotyping over a broad range of DNA amounts. Melt-MAMA genotyping accuracy is not diminished at lower amounts of DNA, even at near-single copy for some assays. The sensitivity of individual melt-MAMAs varies greatly. This *B. anthracis* melt-MAMA (A.Br.003 clade) [4] accurately genotyped DNA regardless of starting amounts as long as it was sufficient to support amplification. (A & B) The respective amplification plots of genomic DNA of ‘G’ allele and ‘A’ SNP allele templates show the amplification curves of templates titrated in ten-fold serial dilutions and in replicates of eight. The number assigned to each amplification curve (1–8) denotes the DNA amount for the starting template. (C) The temperature-dissociation (melt) curve derivatives for all initial template amounts are shown (numbers denote DNA amount shown). This panel illustrates that genotyping accuracy was not affected by DNA amounts, even at near-single copy levels. Similar to TaqMan assays, the detection of low levels of DNA template by Melt-MAMA is also subject to stochastic sampling effects (*B. anthracis* single copy ∼6 fg), which is predictable using a Poisson distribution [4].

**Figure 8 pone-0032866-g008:**
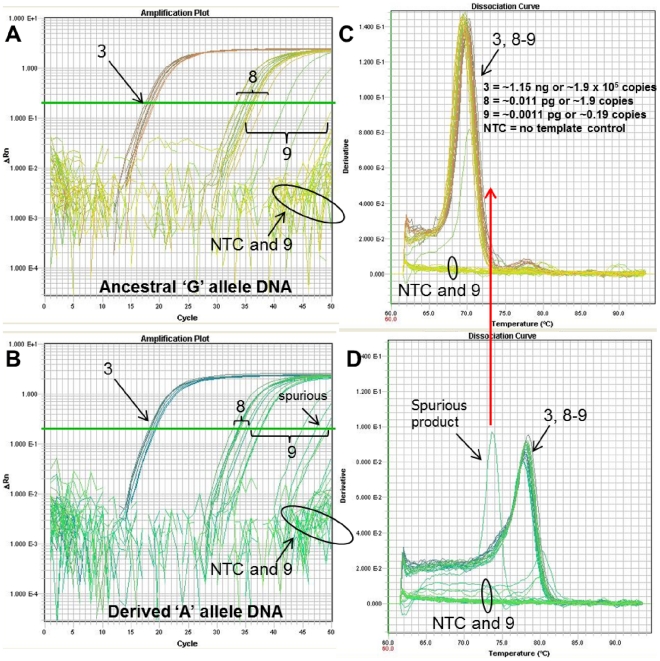
Competition between specific and non-specific amplification at extremely low level DNA amounts. With extremely low-level DNA amounts (<∼2 copies), stochastic, spurious, non-specific amplification could outcompete allele-specific amplification. The *Bacillus anthracis* melt-MAMA targeting the A.Br.003 clade [4] stochastically amplified allele-specific product and non-specific spurious products at amounts of less than a single copy (∼0.19 copies). (A and B) The respective amplification plots of genomic DNA of ‘G’ allele and ‘A’ SNP allele templates show the amplification curves of templates at 1.15 ng and at two low level ten-fold dilution series (near-single copy and less than a single copy) in replicates of eight. The number assigned to each amplification curve (3, 8–9) denotes the DNA amount for the starting template. (C & D) The temperature-dissociation (melt) curve derivatives for the 1.15 ng and lowest template amounts are shown. This panel illustrates that genotyping accuracy was not compromised at DNA template amounts near single copy level, but spurious amplification was observed in dilution points below this level. This spurious amplification had a unique melt-profile that did not match the profile of either allele types (red arrow).

A concern with genotyping assays is whether they are accurate at very low template concentrations, yet to our knowledge this variable has not been extensively tested with Melt-MAMA. Therefore, we investigated the accuracy and sensitivity of Melt-MAMA against a wide range of DNA template amounts using three assays that differentiate six genetic populations of the pathogen *Bacillus anthracis*. Ten-fold serial dilutions of SNP polymorphic allele DNA templates (ancestral and derived, respectively), of equal DNA amounts based on TaqMan assays (Figure 6) and NanoDrop^TM^ measurements were screened on three Melt-MAMAs targeting *B*. *anthracis* lineages A.Br.003, A.Br.004, and A.Br.006 [4]. Dual Probe TaqMan assays are reported to detect DNA templates at single genomic equivalent or copy levels [4], [5], [38] and our TaqMan assay result indicates that levels less than a single copy can be detected in a fashion consistent with stochastic sampling processes (Figure 6). The A.Br.003 Melt-MAMA experiments demonstrated consistent amplification and accurate genotypes for the ancestral and the derived DNA templates at an amount of ∼115 ng–∼11.5 fg (∼1.9×10^7^ to ∼1.9 genome copies; seven-orders of magnitude range) (Figure 7). This Melt-MAMA was one order of magnitude less sensitive than the TaqMan assay (Figures 6 & 7). The A.Br.003 Melt-MAMA displayed consistent amplification and correct genotype calls to low level DNA amounts of 11.5 fg (∼1.9 copies). Below this amount (∼1.15 fg), we observed a combination of correct allele calls, non-specific amplification, or complete amplification failures (Figure 8). For example, a single spurious amplification had a unique melt-profile that did not match the profile of either allele, indicating non-specific amplification (Figure 8D, red arrow). Without close scrutiny of this unique profile, which appears similar to an ancestral genotype profile, it is possible that this profile would be assigned an incorrect allele call. Due to a single spurious amplification observed at < ∼1 copy, and in the event that such spurious amplification remains probable at ∼11.5 fg (∼2 copies), we conservatively consider the lowest limit for this A.Br.003 Melt-MAMA to be 115 fg (∼19 copies) rather than at ∼1.9 copies. Interestingly, all negative controls (n = 34) failed to amplify by 50 cycles (Figure 8, negative controls n = 8; data not shown; negative controls n = 26).

**Figure 9 pone-0032866-g009:**
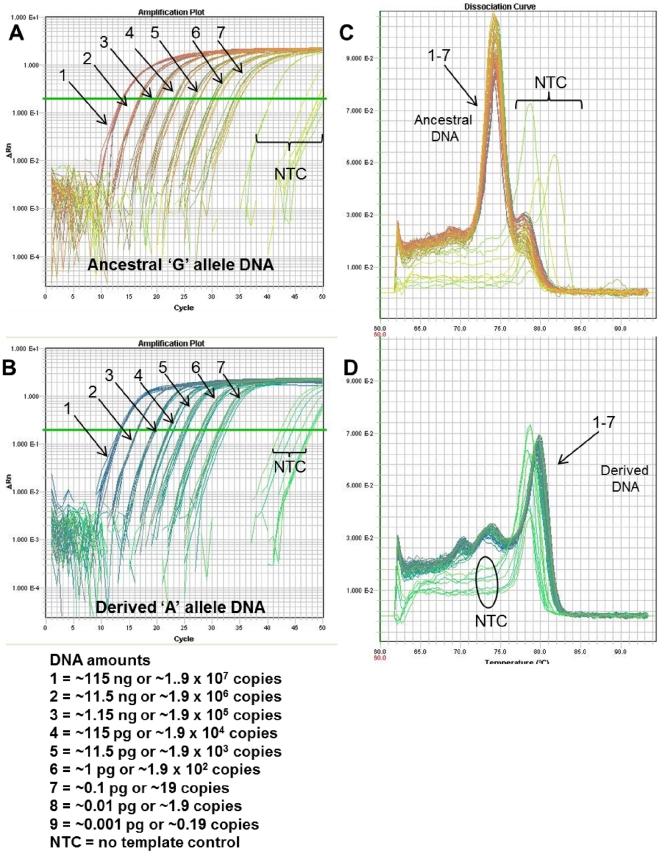
Genotyping over a broad range of DNA amounts. Melt-MAMA sensitivity to low level DNA amounts varies greatly among different assays. *B. anthracis* melt-MAMA targeting the A.Br.006 clade [4] accurately genotyped DNA at amount ∼19 copies. (A & B) The respective amplification plots of genomic DNA of ‘G’ and ‘A’ SNP allele templates show the amplification curves of templates titrated in ten-fold serial dilutions and in replicates of eight. The number assigned to each amplification curve (1–7) denotes the DNA amount for the starting template. (C & D) The temperature-dissociation (melt) curve derivatives for all initial template amounts are shown (numbers denote DNA amount shown). Panels C and D illustrates that genotyping accuracy is obtained across a broad range of DNA template amounts of ∼115 ng to 115 fg. Assay sensitivity to template is limited to ∼19 copies and above. An inherent characteristic of this assay is the occurrence of spurious amplification at extended cycle times (>35) in the absence of template as indicated by the NTCs. Melt-MAMAs detecting low level DNA are subject to stochastic sampling effects (*B. anthracis* single genome copy ∼6 fg), which is predictable using a Poisson distribution [4].

**Figure 10:**
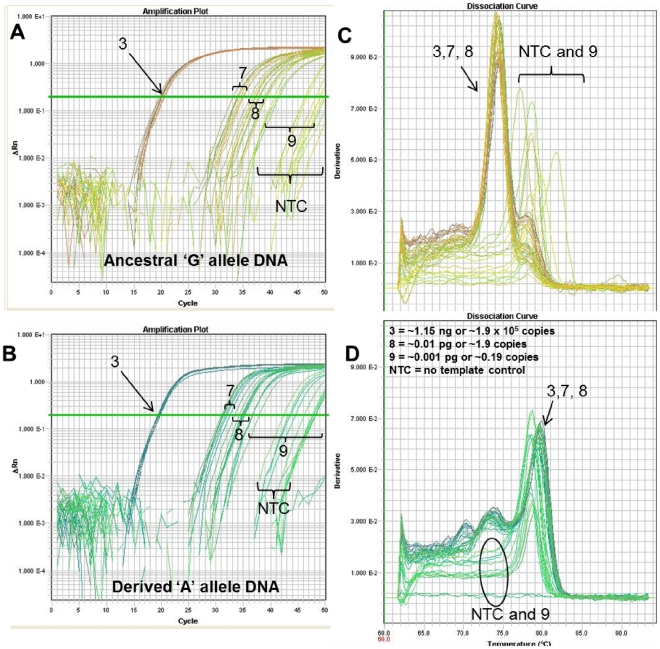
Competition between specific and non-specific amplification at low level DNA amounts. The *B. anthracis* Melt-MAMA targeting the A.Br.006 clade [4] stochastically amplified allele-specific product and non-specific spurious products at amounts of less than ∼19 copies. (A & B) The respective amplification plots of genomic DNA of ‘G’ allele and ‘A’ SNP allele templates show the amplification curves of templates at 1.15 ng and at two low level ten-fold dilution series (∼19 copies and near a single copy) in replicates of eight. The number assigned to each amplification curve (3, 7–9) denotes the DNA amount for the starting template. (C & D) The temperature-dissociation (melt) curves derivatives for the 1.15 ng and lowest template amounts are shown. This panel illustrates that assay sensitivity to template is limited to ∼19 copies and above. Below this template amount, spurious amplification is possible and difficult to differentiate from template-specific amplification.

****Other Melt-MAMAs display less sensitivity to low level DNA amounts. The A.Br.006 [4] Melt-MAMA was two orders of magnitude less sensitive than the TaqMan assay (Figures 6 & 9) and one order of magnitude less sensitive than the A.Br.003 Melt-MAMA (Figures 7, 8, 9, 10). The A.Br.006 Melt-MAMA consistently genotyped allelic templates ranging from 1.9×10^7^ to 19 copies (∼115 ng to ∼115 fg) (six-orders of magnitude range) (Figures 9A & B). At DNA amounts of ≤ ∼11.5 fg (∼1.9 copies), we observed either correct allele calls (Figure 10) or non-specific (spurious) amplification with unique melt-profiles that did not perfectly match the profile of either allele types (Figures 10C & D). We thus consider the lowest limit for this A.Br.006 Melt-MAMA to be at ∼115 fg (∼19 copies) displaying a C_T_ range of 31–35. Most of the negative controls (n = 36) amplified at a C_T_ range of 40–50, generating non-specific products with melt profiles that differed from profiles of the expected two allelic states (Figures 9C & D, negative controls n = 8; data not shown, negative controls n = 26).

Melt-MAMAs and TaqMan assays both can suffer from non-specific spurious background amplification that stochastically occurs when DNA amounts are at extreme low levels (see Figures 8–10). These non-specific amplifications are generated from primers interacting with each other and/or at non-specific regions on the genome template. At prolonged cycle times (35–50), they are detected by SYBR Green, but not in TaqMan assays. This difference in spurious product detection between these two assay types is based on the non-specific nature of SYBR Green and the specific nature of the internal TaqMan probes. SYBR Green binds to all dsDNA which contrasts to TaqMan probes in the Dual Probe TaqMan assays which detects only target product. In light of the probable event of generating spurious product when DNA amounts are extremely low, we are highly conservative with our estimates of assay sensitivity. We routinely exclude products with amplification greater than 32 C_T_ values. We discard assays that show amplification of negative controls with C_T_ values less than 35. We emphasize the importance of careful scrutiny of all data to assure the exclusion of products from the rare spurious amplification and the inclusion of positive template controls with every experiment.

### Sensitivity can depend on allelic template type for some Melt-MAMAs

The sensitivity of A.Br.003 and A.Br.006 assays were equal regardless of the allelic state of the template (Figures 7–9A & B). This characteristic is not true for all Melt-MAMAs. The sensitivity limit of A.Br.004 assay depends on which allelic template is in the reaction. The A.Br.004 ancestral AS-MAMA primer consistently amplified and genotyped ∼1.9 copies (11.5 fg) of ancestral template, whereas the derived AS-MAMA primer with the same amount of derived DNA template (1.9 copies or ∼11.5 fg) failed or generated spurious products (indicated by unique melt-profiles, data not shown). The derived AS-MAMA primer consistently amplified and accurately genotyped ∼19 copies of derived template. These differences indicated that the ancestral AS-MAMA primer has one order of magnitude more sensitivity to the ancestral template than the derived AS-MAMA primer has for the derived template (∼1.9 copies and ∼19 copies, respectively). Despite the sensitivity differences among AS-MAMA primers for their respective allelic templates, the efficiency differential between the AS-MAMA primers for the identical template remain firmly intact. For accurate SNP genotyping capability, the ancestral AS-MAMA primer would have greater efficiency with the amplification of the ancestral DNA template compared to the derived AS-MAMA primer for this same ancestral DNA template. Likewise, the opposite is true. For example in the A.Br.004 assay, if ∼2 copies of a derived template is in the reaction (an amount where this assay fails), the derived AS-MAMA primer lacks the sensitivity for this copy amount by one order of magnitude and the ancestral AS-MAMA primer lacks the sensitivity for this amount of derived template by ∼2 orders of magnitude. The event of mis-genotyping DNA at extremely low amounts remains improbable due to this efficiency differential.

### Two-Locus (duplexed) Melt-MAMA

**Figure 11:**
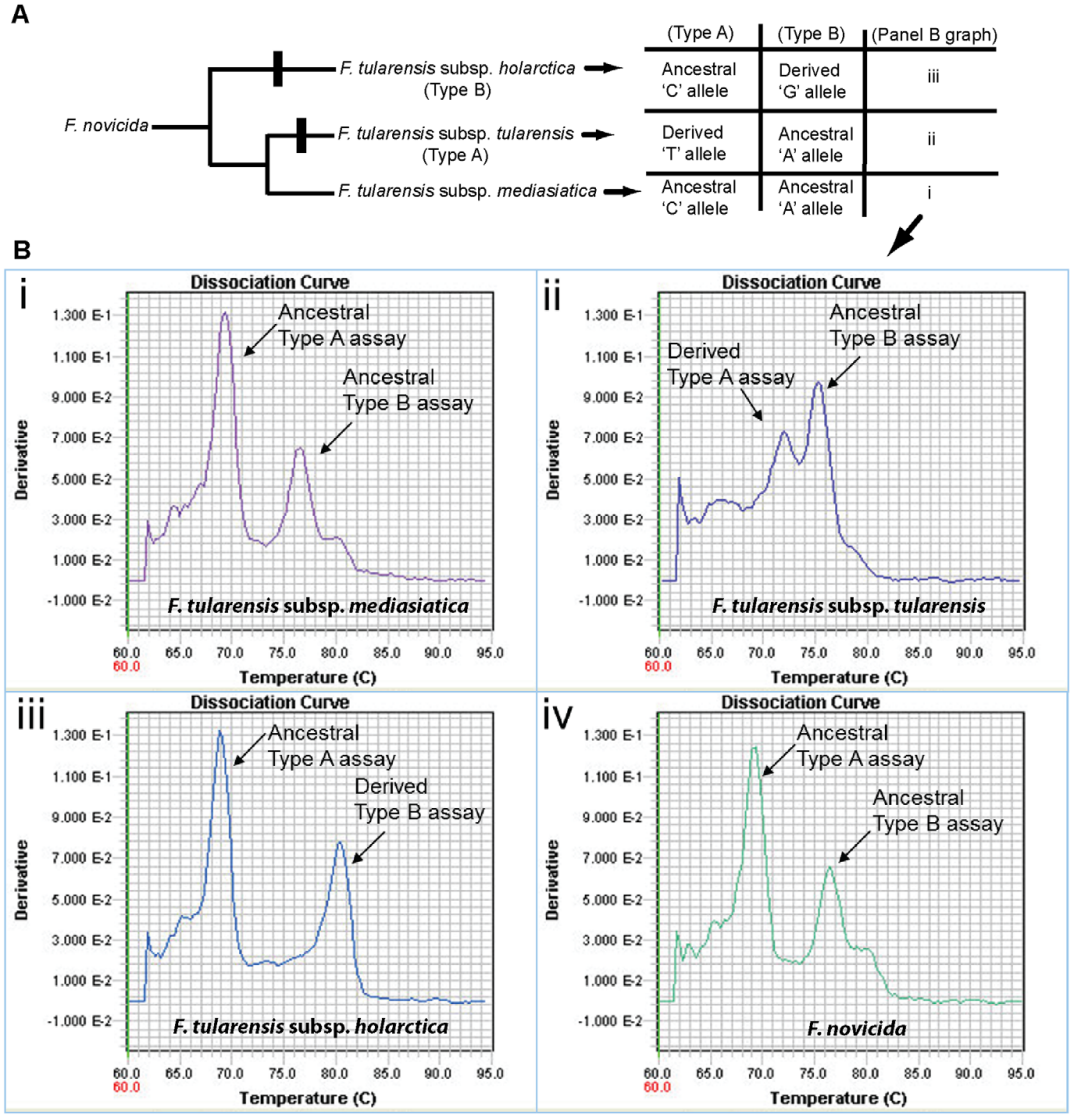
Two-Locus (duplexed) Melt-MAMA development. (A) A phylogenetic topology of the three subspecies of *F. tularensis* rooted with *F. novicida*. The SNP-signatures specific for the two pathogenic subspecies of *F. tularensis* (indicated by black bars) were incorporated into Melt-MAMAs. The table (right) indicates expected allele states (derived and ancestral) for strains from each *F. tularensis* subspecies represented on the topology; *F. novicida* would have the same allelic states as *F. tularensis* subsp. *mediasiatica*. (Bi–iv) Temperature-dissociation (melt) curves (derivative) of allele-specific PCR products from *F. tularensis* strains amplified in the duplexed assay (Type A and Type B). Each profile show two melt-curve peaks generated from a single *F. tularensis* strain. Each peak corresponds to the allele-specific PCR product for a single SNP-locus in the duplexed assay.

To further enhance the cost-effectiveness of Melt-MAMAs, we developed a two-locus duplex Melt-MAMA targeting two different SNPs that define two clinically relevant subspecies within *F. tularensis* (*F. tularensis* subsp. *tularensis* and *F. tularensis* subsp. *holarctica*). Four *Francisella* strains – representing three different *F.* *tularensis* subspecies and the genetic near neighbor *F. novicida* (Figure 11A), were genotyped to demonstrate the utility of this assay. Each strain showed clear, unambiguous melt-dissociation profiles that matched the expected genotype for each respective strain (Figure 11Bi–iv). Additional screening across a small panel of geographically and genetically diverse set of *F. tularensis* isolates comprised of high quality genomic DNA resulted in perfect agreement with genotypes obtained from single-plex assays and multi-locus variable number of tandem repeats assay (MLVA) [39] (data not shown). This 100% agreement between independent approaches demonstrates the proof of concept that duplex Melt-MAMAs are effective genotyping assays and a viable cost-saving strategy. We estimate that the consolidation of two SNP markers into a single Melt-MAMA duplex assay reduced reagent usage and labor by ∼50%.

### Allele-Specific MAMA genotyping based upon agarose sizing

**Figure 12:**
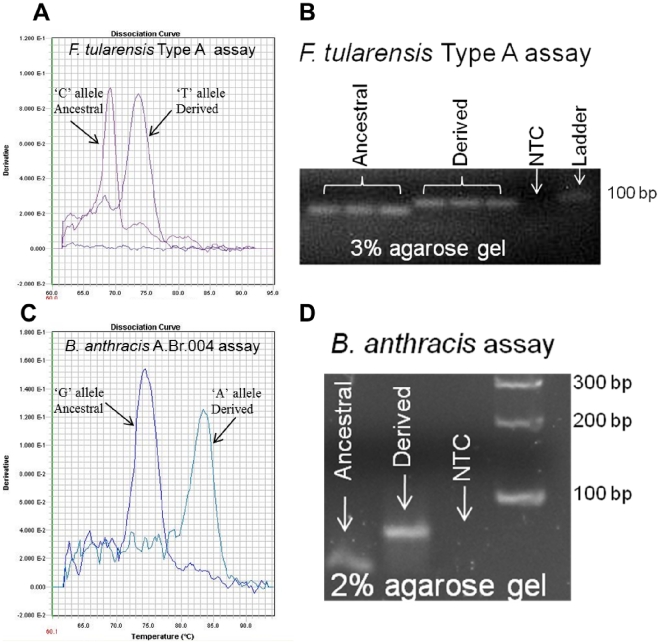
Allele-Specific MAMAs Based Upon Agarose Sizing. Allele-specific SNP MAMAs from two pathogen species were transferred from a real-time PCR instrument (A & C; Melt-MAMA) to conventional PCR coupled with agarose gel electrophoresis (B & D; Agarose-MAMA) under identical assay conditions. Genotypes from Melt-MAMAs are determined by temperature-dissociation (melt) curves, whereas genotypes from Agarose-MAMAs are determined by the amplicon size differences.

Genotyping samples using Melt-analysis requires somewhat sophisticated real-time PCR instruments, which come at a heavy upfront cost and require technically trained personnel. Because the Melt-MAMAs presented here are based exclusively on unlabeled primers of distinctly different lengths, as expected, we determined that our assays are amenable to the lower technological approach of conventional PCR coupled with agarose-gel electrophoresis platform (Figure 12). We obtained these results using primer ratios and template concentrations identical to those used on real-time instruments. For some of our assays, we adjusted the annealing temperature from 60 to 55°C, which improved the amplification and detection on agarose gels. We believe this was due to the difference in MgCl_2_ concentrations between SYBR Green master mix (Applied Biosystems, Foster City, CA) and our conventional PCR master mix (see methods). Commercially pre-made SYBR Green master mixes typically use high concentrations of MgCl_2_ to overcome the inhibitory property of SYBR Green dye [40]. On an agarose-gel platform, we resolved allelic differences through the differential amplicon size made possible by the 19 bp GC-clamp label. PCR products generated by real-time PCR from two different pathogen assays, *F. tularensis* type A-specific and a *B. anthracis* A.Br.004-specific, were viewed by Melt-analysis (Figures 12A & C). PCR products from these same assays were generated by conventional PCR, under identical reaction conditions, and visualized on a 3% (Figure 12B) and 2% agarose gel (Figure 12D), respectively. Allele-type was differentiated based on amplicon size differences, which were clearly distinguishable (Figures 12B & C). An additional set of 22 *B*. *anthracis* AS-MAMAs (initially developed as Melt assays; see subset in Table S1), tested by conventional PCR and genotyped by agarose sizing (data not shown) demonstrated the same results shown in Figure 12.

The successful development of AS-MAMAs on an agarose-gel platform [29], [30] and on capillary electrophoresis (CE) [41] have been reported. On an agarose-gel platform the allele-specific size differences are achieved by the incorporation of an AT-rich 5′ tail to one of the AS-MAMA primers to provide added length. In contrast, on the CE platform allele-specificity is based on differential sequences encoded at the 5′tail of each AS-MAMA primer. This facilitates the differential incorporation of allele specific fluorescent dyes, which can be subsequently visualized on CE, a technology termed as CE universal tail MAMA (CUMA) [41]. We developed AS-MAMAs that function equally well on real-time instruments and conventional PCR coupled with agarose sizing under identical PCR conditions. The only exception was the need to reduce the annealing temperature in some assays due to master mix differences. In theory, Melt-MAMA could be transferred to CE platform since this too is principally based on conventional PCR. Our single attempt of Melt-MAMA to CUMA conversion resulted in success indicated by the retention of accurate genotyping (data not shown). The amenability of assays across these multiple platforms would allow assay technology to transfer between research facilities without additional redesign or extensive assay optimization. This is particularly valuable as genotypic data from rudimentary public health labs can be integrated readily with databases generated in technically advanced genome centers. High capacity screening is not needed in many regional situations, but the results need to be directly comparable to global databases.

### Synthetic allele-specific templates

Every AS-MAMA experiment must include genotyping of positive control templates possessing the derived and ancestral SNP states. This poses a challenge in that access to DNA templates that can appropriately serve as positive controls for a given assay may not be possible for every laboratory. This problem could hamper the assay technology transfer between collaborating research facilities. To address this issue, we have developed an approach to create SNP-polymorphic synthetic positive controls for any given AS-MAMA (See Figure S1 and Table S1). These synthetic positive controls are PCR amplicons generated by allele-specific unlabeled primers and they can be performed in-house within any research facility capable of conventional PCR (see methods). We successfully generated synthetic positive control templates for 20 assays that serve as both Melt-MAMAs and Agarose MAMAs (Table S1).

In conclusion, Melt-MAMAs are highly effective SNP genotyping tools that are approximately fourteen times less expensive than Dual Probe TaqMan assays. The studies presented here identified specific strategies and work flow (Figure 5) that effectively increased assay success rate at the first design attempt, providing performance that rivals the success rate of Dual Probe TaqMan assays. These specific strategies include 1) designing the destabilizing mutation to have a unique base character that differs from the base character of the genomic DNA template and the alternate AS primer and 2) altering the ratios of AS primer concentrations to correct poorly genotyping assays. We also described the size advantage of the GC-clamp label that permits amenability of identical assays on two frequently used but very different instrumentation platforms. Data presented here advanced our knowledge of the capacities and design principles of Melt-MAMAs, which will maximize the successful application of this technology throughout the wider scientific community.

## Methods

### Melt-MAMA Design

All Melt-MAMAs used in this study were designed using Primer Express 3.0 software (Applied Biosystems, Foster City, CA) or a free on-line primer design software by Premier Biosoft (http://www.premierbiosoft.com/netprimer/). Assay design consisted of four features: 1) Two AS forward primers, where the 3′ ends correspond to one of the allele-states for the SNP locus, and a common reverse primer, 2) a deliberate ‘destabilizing’ mismatch at the penultimate or antepenultimate base position (–2 or –3, respectively from the 3′-end), 3) a GC-clamp at the 5-end of one of the allele-specific forward primers (12–19 bp consisting of a GGGGC repeat pattern), and 4) a small PCR product (60 bp–100 bp) (Figures 1 & 2). The GC-clamp served as a label that would permit easy differentiation between the two allele-specific PCR products, based on differences in GC content. This differentiation is based on dissociation curves (melt analysis) [17], [20], [21] (Figures 3B & 3D). Typically the “derived” AS-MAMA primer was labeled with the GC-clamp. Each Melt-MAMA reaction mixture (7 µl or 10 µl) contained 1x SYBR Green PCR master mix (Applied Biosystems, Foster City, CA), two forward AS primers, a common reverse primer, and 1 µl of DNA template at ∼1ng/µl. DNA templates were produced from Whole Genome Amplifications (WGA; Qiagen, Valencia, CA) or genomic DNA extracted by various types of preparations (standard heat soak or chloroform preparations). Melt-MAMAs were performed on an Applied Biosystems 7900HT Fast real-time PCR system with SDS v2.3 software. Thermocycling parameters were 50°C for 2 min, 95°C for 10 min, and then cycled at 95°C for 15s and 55°C–60°C (dependent on assay; see Table S1) for 1 min for 33 cycles. End-point PCR products were subject to melt analysis using a dissociation protocol comprising 95°C for 15 s, followed by incremental temperature ramping (0.2°C) from 60°C to 95°C. SYBR Green fluorescent intensity is measured at 530 nm at each ramp interval and plotted against temperature. Melt-MAMAs (n = 185) were designed by multiple research teams targeting eight diverse bacterial pathogens (Table 1). A subset of Melt-MAMAs (targeting different genetic groups of *F. tularensis* and *B. anthracis*) were used to illustrate key functional aspects of this technology (Table S1). The SNP signatures in *F. tularensis* specific assays targeted sublineages within the B.Br.013/014 subclade (Birdsell & Keim, unpublished data) and the SNP signatures in *B. anthracis* assays targeted the major lineages previously described [4].

### Assay optimization through altering the primer ratios

To efficiently determine the optimized condition for each assay, a standard work flow approach for assay validation was followed (Figure 5). Initial validation included three different reactions consisting of three ratios of AS-MAMA primers (ancestral and derived, respectively) at 1∶1, 4∶1, and 1∶4 run at a 60°C annealing temperature. Adjustments for subsequent rounds of assay validation were determined following the initial results. Readjustments included the less or more severe ratio alteration of the AS-primers (2∶1 and 1∶2; 6∶1 and 1∶6) or testing at lower or higher annealing temperatures (55°C–62.5°C). Assays were deemed valid when they accurately and consistently genotyped both allelic DNA templates under identical conditions.

### Design success rates of assays

To measure the successful design rate of Melt-MAMAs, we recorded the number of successful assays after the first design attempt on 185 assays targeting eight diverse bacterial pathogens (Table 1). Assays that successfully functioned after primer ratio alteration were included in this category. To determine if design success rate is dependent on genome size and GC content of the target organism, we recorded the design success rate of assays per target organism with discrete genome size and GC content (Table 1).

### Assay Robustness and Sensitivity

We tested the sensitivity and genotyping accuracy (robustness) of a small subset of Melt-MAMAs (three *B. anthracis* specific) across a broad range of DNA concentrations (115 ng–1.15 fg) by performing 10-fold serial dilutions in replicates of eight (Figures 6–10).

### Duplex Melt-MAMA

Two *F. tularensis* assays specific to different genetic groups (*F. tularensis* subsp. *tularensis* and *F. tularensis* subsp. *holarctica*) were combined into a single duplex assay (Figure 11; Table S1). To permit the proper dissociation temperature (*T*_m_) spacing of all AS-PCR products in this duplex assay a GC-clamp was added on to the common reverse primer of the *F. tularensis* subsp. *holarctica* assay in addition to one of the allele-specific MAMA primer (See assay design on Table S1). In contrast, the *F. tularensis* subsp. *tularensis* assay contained a GC-clamp only on one of the allelic-specific MAMA primers (Table S1). To fully validate the duplex assay, we screened it across a small panel of geographically diverse *F. tularensis* DNA samples (data not shown).

### Agarose MAMA

We converted a subset of our Melt-MAMAs to a lower technology platform using conventional PCR coupled with agarose gel electrophoresis (Agarose-MAMAs) (Table S1). *F. tularensis* subsp. *tularensis* and *B. anthracis* A.Br.004 Melt-MAMAs were used to illustrate this size-based genotyping method (Figure 12). Selected assays possessed a 19 bp GC-clamp to provide sufficient size difference among AS-PCR products visible on 3% and 2% agarose gels. The conventional PCR master mix comprised two forward AS primers and a common reverse primer starting at 0.2 µM (IDT, San Diego, CA), 1x PCR buffer without MgCl_2_ (Invitrogen, Carlsbad, CA), 2 mM MgCl_2_ (Invitrogen, Carlsbad, CA), 200 µM of each dNTPs (Invitrogen, Carlsbad, CA), 0.8 units of Platinum *Taq* DNA polymerase (Invitrogen, Carlsbad, CA), 1 µl of template at ∼1ng/µl, and molecular grade water to a final volume of 10 µl. Agarose-MAMAs were performed under the following conditions: PCR amplifications were conducted on a MJ Research 96 well block thermal cycler DNA engines equipped with hot bonnets. PCRs were raised to 94°C for 5 min to denature the DNA and to activate the hot-start *Taq* DNA polymerase, then cycled at 94°C for 30 sec, 55°C–62.5°C (depended on assay) for 30 sec, 72°C for 30 sec, with a final extension at 72°C for 5 min. The PCR amplicons and a 100 bp ladder (Invitrogen, Carlsbad, CA) were electrophoresed at 100 V for 90–110 minutes on a 2% or 3% agarose gel (Fisher Scientific, Pittsburgh, PA) prepared in 1x TAE and visualized with SYBR Safe (Invitrogen, Carlsbad, CA).

### Synthetic Positive Control templates

Each allele-specific synthetic positive control is an amplicon generated by conventional PCR using the same starting template (Figure S1). The allele SNP-state of the synthetic positive control is constructed by the base identity of the 3′ end of the forward primer. This is the reason why the same starting genomic DNA template can generate synthetic positive controls possessing either SNP states. Each allele-specific amplicon was generated by the use of an allele-specific forward primer and a common reverse primer (IDT, San Diego, CA). PCR cycled at 94°C for 30 sec, 58°C for 30 sec, 72°C for 30 sec, with a final extension at 72°C for 5 min (total of 35 times). All reagents and other PCR conditions were identical to those cited in the Agarose MAMA methods. These final PCR amplicons, possessing the ancestral and derived SNP-states, were used as positive control templates for their specific assay at 1/100,000 dilution.

## Supporting Information

Figure S1 **Design principle of the allele-specific synthetic positive control.** This figure shows the construction of allele-specific amplicons (A & B). The SNP allele state of the amplicon is encoded by the base identity of the 3′ end of the allele-specific (AS) forward primer in the PCR (Ai–iii & Bi–iii). The annealing of AS- primers to their allelic (matched) template (Ai) and non-allelic (non-matched) template (Bi) is shown. (Aii & Bii) *Taq* Polymerase extends from the 3′ end of the AS-MAMA primer on both allelic and non-allelic templates. The single 3′-end mismatch does not significantly hinder the *Taq* Polymerase-mediated extension on the non-allelic template (Bii). The newly synthesized DNA made in the previous PCR step (Aii & Bii) serves as the template for amplicon replication in the second PCR cycle (Aiii & Biii). This results in the formation of a perfect primer-template complex on the non-allelic template PCR (Biii). Maximal PCR efficiency is achieved for both allele-specific amplicon types (Aiv & Biv). Each amplicon is designed to be of ∼150–200 bp length.
(DOCX)

Table S1 **Primer sequences and PCR conditions for Melt-MAMAs.** Primer sequences for synthetic positive controls are provided for a subset of assays.
(XLSX)
