# Supplementary figures and images for: Melt Analysis of Mismatch Amplification Mutation Assays (Melt-MAMA): A Functional Study of a Cost-Effective SNP Genotyping Assay in Bacterial Models
Source: PLoS One. 2012 Mar 16;7(3):e32866. doi: 10.1371/journal.pone.0032866 (PMC3306377; doi:10.1371/journal.pone.0032866)

**Figure S1**


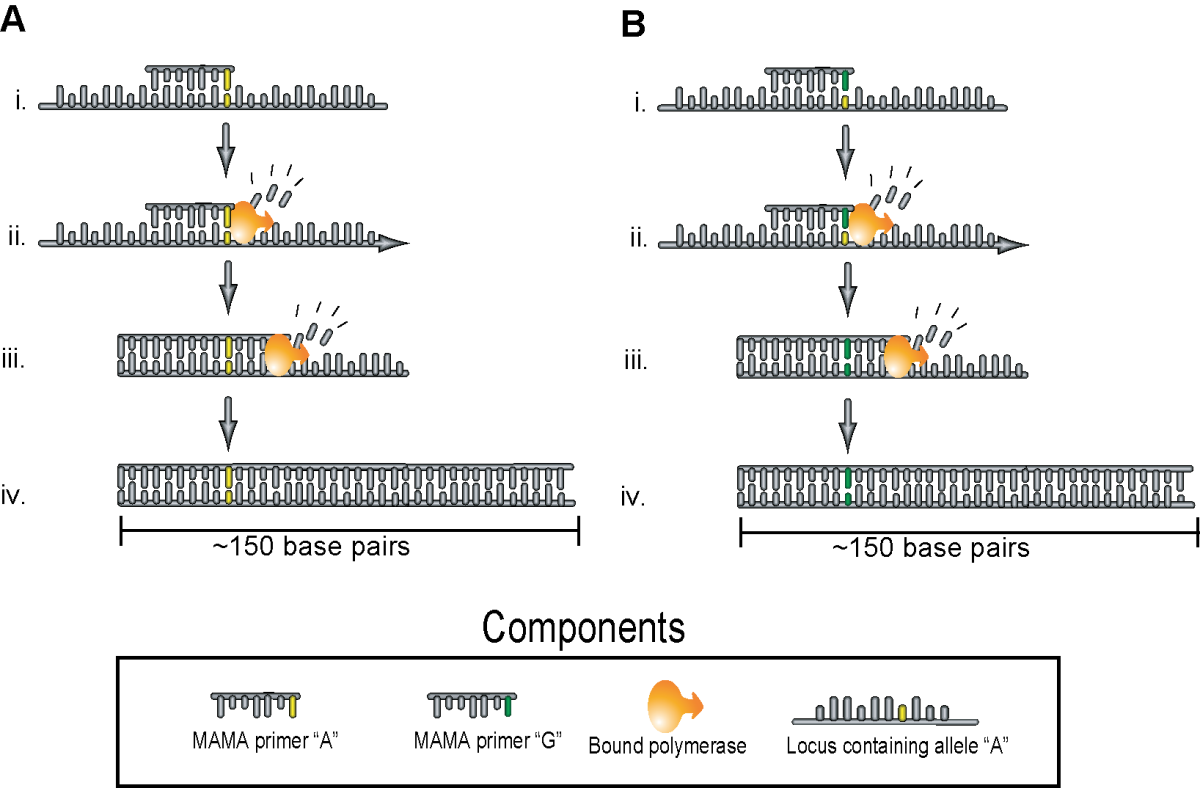

Supplement: Figure S1 — Design principle of the allele-specific synthetic positive control. This figure shows the construction of allele-specific amplicons (A & B). The SNP allele state of the amplicon is encoded by the base identity of the 3′ end of the allele-specific (AS) forward primer in the PCR (Ai–iii & Bi–iii). The annealing of AS- primers to their allelic (matched) template (Ai) and non-allelic (non-matched) template (Bi) is shown. (Aii & Bii) Taq Polymerase extends from the 3′ end of the AS-MAMA primer on both allelic and non-allelic templates. The single 3′-end mismatch does not significantly hinder the Taq Polymerase-mediated extension on the non-allelic template (Bii). The newly synthesized DNA made in the previous PCR step (Aii & Bii) serves as the template for amplicon replication in the second PCR cycle (Aiii & Biii). This results in the formation of a perfect primer-template complex on the non-allelic template PCR (Biii). Maximal PCR efficiency is achieved for both allele-specific amplicon types (Aiv & Biv). Each amplicon is designed to be of ∼150–200 bp length. (DOCX) [file pone.0032866.s001.docx]
